# Risk and protective factors associated with depression in young people: what do Nigerian university students know?

**DOI:** 10.4314/ahs.v22i2.67

**Published:** 2022-06

**Authors:** Chibueze Anosike, Maxwell O Adibe, Nneka U Igboeli, Uchechukwu E Onwukwe, Chikaodili L Okwume

**Affiliations:** 1 Department of Clinical Pharmacy and Pharmacy Management, University of Nigeria, Nsukka; 2 Pharmacoeconomics and Mental Health Research Group, University of Nigeria, Nsukka; 3 Rommar Pharmacy, Ikorodu, Lagos State, Nigeria; 4 Cleon Pharmacy, #127 Park avenue, GRA, Enugu, Nigeria

**Keywords:** Depression, risk, protective factor, knowledge, university students, Nigeria

## Abstract

**Background:**

University students appear to experience a significantly higher rate of depression compared to the general population. However, there is limited data showing how much Nigerian university students know about the risk and protective factors related to depression.

**Objectives:**

To assess the knowledge of risk and protective factors associated with depression in young people among students of a Nigerian university.

**Methods:**

A cross-sectional descriptive survey was conducted among simple randomly selected students of the University of Nigeria, Nsukka. Two validated self-administered questionnaires were used for data collection. Descriptive statistics and multivariate binary logistic regression were used for the data analysis.

**Results:**

Out of 1591 participants, about 47% and 60% had good knowledge of risk and protective factors related to depression, respectively. The course of study, year of study, contact with a depressed person, and personal experience of depression significantly predicted students' knowledge of risk factors for depression. Similarly, course of study, year of study, and gender were the significant predictors of students' knowledge of protective factors against depression.

**Conclusions:**

The students had good knowledge of protective factors against depression, but were poor in knowledge of its associated risks. Therefore, provision of mental health services in the universities is recommended.

## Introduction

Depression is a significant global public health problem, even though it is often neglected and untreated among sufferers.^1^ In 2015, the World Health Organization (WHO) statistics showed that about 350 million people around the world are estimated to suffer from depression. 2 Depression is one of the major causes of nearly one million cases of suicides yearly.^1^,^2^ Depression is a recurrent mental health problem, usually characterized by a diminished quality of life, reduced work productivity, poor academic performance, and suicidal ideation.^3^ Depression contributes substantially to the global burden of disease. This year 2020, depression is the second-highest cause of disability worldwide.^4^ Depressive disorders are among the most common form of mental illness in young people.^5^ Around the world, depression-induced suicidal behaviour is ranked among the primary cause of death in young people.^6^, ^7^ The high prevalence of depression-induced suicides could be seen in young people in both developed and developing countries, particularly among college and undergraduate students.^8^

In Africa, university students appear to experience a significantly higher rate of depression relative to the general population.^8^ Previous studies in Africa reported a prevalence of depression among undergraduate university students ranging from 10% to 85%.^8^, ^9^ Nevertheless, a cross-sectional study among medical students in a Nigerian university reported the prevalence of depression to be 23.3% 10. In the literature, several factors predisposing young people to depression are well documented. The factors may include higher study year, older age,^8^,^11^ female gender,^12^, ^13^ lower socioeconomic status,^8^, ^11^ stressful and traumatic life events,^13^ witnessing parental violence,^14^ chronic illness, addictive behaviour,^13^ physical inactivity, 15 lack of social support,^16^ and poor academic performance.^17^

Conversely, preventive interventions target the modification of factors that predispose individuals to depression. It aims to strengthen the coping mechanisms of individuals who are at risk of depression.^18^ Effective interventions among university students would require the identification of causal risk factors for depression.^19^ The knowledge of protective factors would be beneficial in the fight against depression. It is generally known that positive relationships, social support, adequate physical exercise, proper nutrition, sufficient and regular sleepig pattern, and avoidance of illicit drug use may help young people avert or deal successfully with depression.^20^–^22^ Therefore, ascertaining what university students know about risk and protective factors linked with depression in young people would serve as a guide for future public health educational interventions. Therefore, this study aimed to assess what Nigerian university students know about the risk and protective factors connected with depression in young people and assess the demographic predictors of students' knowledge.

## Methods

### Study design and location

This research was a cross-sectional survey conducted among undergraduate students of the University of Nigeria, Nsukka. The institution presently has seventeen faculties. Science-based faculties were (1) Agriculture, (2) Basic Medical Sciences, (3) Biological sciences, (4) Dentistry, (5) Engineering, (6) Environmental studies, (7) Health sciences, (8) Medicine, (9) Pharmaceutical sciences, (10) Physical sciences, (11) Veterinary Medicine, and (12) Social sciences. However, the non-science-based faculties were (1) Arts, (2) Business administration, (3) Education, (4) Law, and (5) Vocational Technical Education. The university has a balanced mix of a student population from various ethnic, cultural, and religious backgrounds.

### Ethical considerations

The ethical approval for this study was obtained from the National Health Research and Ethics Committee on August 14, 2019, with the approval number: NHREC/05/01/2008B-FWA00002458-IRB00002323. A written informed consent was obtained from all participants before study initiation. Students who refused consent were asked to state the reason(s) for the decision. To ensure privacy and confidentiality, the responses were anonymous and no personally identifiable information was collected.

### Sample size and sampling technique

The sample size for this study was calculated using the Raosoft® online sample size calculator. As at the time of the study, the total undergraduate student population of the university was 38,532. Thus, assuming a 5% margin of error and a 95% confidence interval, the minimum sample size required for the study was 381. However, 1,635 students were invited to participate in the study to increase the statistical power of the study. The participants were drawn proportionately by a computer-generated simple random sampling across various faculties and departments of the university using the official class list.

### Study instruments

In this study, two self-administered paper-based questionnaires were used to assess students' knowledge of the risk and protective factors associated with depression in young people. The questionnaires were the Knowledge of Risk Factors for Teen Depression Questionnaire (KRFD-Q) and the Knowledge of Protective Factors against Teen Depression Questionnaire (KPFD-Q).^23^ The questionnaires were developed and validated after a thorough literature search, expert consultations, and following appropriate validation protocols. The questionnaires, KRFD-Q and KRFP-Q, were satisfactorily reliable with Cronbach alpha of 0.72 and 0.71, respectively. The test-retest reliability of the KRFD-Q (r = 0.83, p < 0.001) and KPFD-Q (r = 0.77, p < 0.001) were within acceptable limits. Both questionnaires have “true,” “false,” and “not sure” responses to each item. The correct answers were “true” for some questions and “false” for others. However, some items were negatively worded and thus reversed during data analysis. The items 1, 5, 8, 10, and 15 in the KRFD-Q were negatively worded, while items 3, 4, 7, 9, and 15 were negatively worded in the KPFD-Q. In scoring the instruments, correct answers were assigned “1,” while the incorrect answers were assigned “0”. All “not sure” responses were assumed incorrect. Additionally, an attached form was used to obtain student's demographic data such as faculty, department, year of study, age, marital status, and residence. Four questions were included to capture students' attitudes and practices (experiences) toward depression. The questions were, “(1) Have you ever had contact with someone with depression?; (2) Have you ever visited a psychiatric hospital to see someone diagnosed with depression?; (3) Do you know of any family member or friend who suffers from depression?; and (4) Have you ever experienced depression?” The students' knowledge was categorized into good and poor based on whether they are above or below the mean population score.

### Data collection procedure

The data were collected in batches after the usual lecture and practical classes, where applicable. The students were first briefed by the lead researcher on the study protocols and objectives. A unique study identification code was created and appended to all the questionnaires. Subsequently, the questionnaires were distributed to selected students who gave written informed consent to participate in the study. The questionnaires were retrieved immediately after completion by the researcher and their assistants. The researchers and their assistants were available to ensure that participants independently responded to each item. The data file in Microsoft Excel was encrypted as it was transferred between the research team to prevent unauthorized access to the file. The data for this study were collected between September 2 to October 31, 2019.

### Statistical analysis

Descriptive statistics (frequency, percent) was used to summarize students' demographic variables and knowledge scores. Pearson correlation was performed to determine the relationship between independent and dependent variables. Multivariate binary logistic regression was used to determine the independent predictors of students' knowledge of risk and protective factors linked with depression in young people. The adjusted odds ratio (AOR) of the independent variables were calculated. Probability values (p-values) less than 0.05 were considered statistically significant. All data analysis was performed using IBM Statistical Products and Services Solution (SPSS) version 20 for Windows software.

## Results

### Students' demographic characteristics

Table 1 showed the demographic characteristics of the participants. Overall, 1591 students participated in the study, giving a response rate of 97.3%. More than half of the students were female (54.4%) and were in science-based disciplines (58.4%). Approximately half of the students (51.7%) were aged 21 to 25 years. About 59% of the participants have had contact with a depressed person, and 45.1% had experienced depression.

**Table 1:** Students' demographic characteristics (n = 1,591)

Variables	Frequency*	Percent
**Course of study**		
Sciences	930	58.4
Non-sciences	661	41.5
**Year of study**		
First-year	396	24.9
Second-year	383	24.0
Third-year	337	21.2
Fourth-year	352	22.1
Fifth-year	123	7.7
**Gender**		
Female	866	54.4
Male	725	45.5
**Age (years)**		
16–20	631	39.6
21–25	824	51.7
26–30	110	6.9
≥ 31	15	1.0
**Marital status**		
Single	1533	96.2
Married	52	3.3
**Residence**		
Hostel	808	50.7
Off-campus	779	48.9
**Contact with a depressed person**		
Yes	923	58.5
No	656	41.5
**Ever visited a depressed person in a** **psychiatric hospital**		
Yes	173	10.9
No	1414	88.8
**Knew any family member or friend who** **suffers from depression**		
Yes	536	33.6
No	1051	66.0
**Personal experience of depression**		
Yes	718	45.1
No	866	54.4

* Some data were missing

### Knowledge of risk factors for depression in young people

Table 2 showed the knowledge scores of the participants about the risk factors associated with depression in young people. Almost 90% of the students knew that parental neglect of a child might lead to depression. At least 77% of the students had correct knowledge that family conflict, low self-esteem, and stressful life events increase the risk of depression. Fewer than 33% of the participants correctly identified depression to be more common in females compared to male teenagers and young adults. Overall, 67.2% was the mean score of the student population and 47.3% had good knowledge of the risk factors for depression in young people.

**Table 2:** Students' knowledge of risk factors for depression in teenagers and young adults

SN	Questions	Correct (n %)
1	People whose parents abuse or misuse alcohol and other psychotropic substances have little or no risk of having depression	856 (53.7)
2	Poor parenting (e.g., neglect of a child's emotional needs) may lead to depression in adolescents	1423 (89.4)
3	The victims of abuse and maltreatment are more likely to be depressed	1454 (91.3)
4	Those whose family members are not at peace with one another are more likely to suffer depression	1235 (77.5)
5	The relationship with peers does not influence the risk of depression	866 (54.4)
6	Depression is more common among people with low self-esteem and perceived incompetence	1257 (78.9)
7	Individuals whose parents suffer from depression are more likely to experience depression also	736 (46.2)
8	The loss of a loved one cannot result in depression	841 (52.8)
9	Stressful events of life (e.g., chronic sickness, accident) increases the likelihood of depression	1271 (79.8)
10	Poor social skills (e.g., inability to make friends) decreases the risk of depression	991 (62.2)
11	A student is more likely to be depressed if his/her academic performances are poor	1320 (82.9)
12	Individuals with little means of livelihood are at higher risk of Depression	1074 (67.4)
13	Teen depression is more common in females compared to males	517 (32.5)
14	Marital conflict worsens the incidence of depression among family members	1206 (75.7)
15	Lack of support from family and friends does not affect the risk of depression	989 (62.1)

Mean knowledge score = 67.2%

### Knowledge of protective factors against depression in young people

Table 3 contained the students' knowledge scores of the protective factors against depression in young people. About 83% of the students are aware that empowerment policies and programs targeted at poverty alleviation would reduce the risk of depression. Nearly half of the participants (46.3%) have correct knowledge that avoiding social stigma would minimize the risk of depression. More than half of the students (67.3%) knew that proper nutrition was essential in relieving physical and emotional stress owing to depression. Less than 38% of the students identified that avoiding alcohol abuse and acquiring excellent communication and language skills minimizes the probability of being depressed. About 40% of the students recognized that a sufficient and regular sleep cycle helps in coping with depression. Overall, the students' population mean score was 62.1% and more than half of the students (59.5%) had good knowledge of the protective factors against depression in young people.

**Table 3:** Students' knowledge of protective factors against depression in teenagers and young adults

SN	Questions	Correct (n %)
1	Policies and programs targeted at empowering poor people will reduce the problem of depression	1319 (82.8)
2	Improved education and childcare prevent people from being depressed	1304 (81.9)
3	Socially disadvantaged teenagers are less likely to be depressed if they are stigmatized	738 (46.3)
4	People who are well-informed of depression are less likely to cope with the disorder	683 (42.9)
5	If schools prohibit bullying and harassments, students will less likely feel depressed	1146 (71.9)
6	Good nutrition relieves physical and emotional stress owing to depression	1072 (67.3)
7	Avoidance of substance abuse does not make depressed persons feel all right	594 (37.3)
8	Improving attention and memory of a depressed person may initiate the quick recovery	1203 (75.5)
9	Learning good communication and language skills has nothing to do with depression	581 (36.5)
10	Having good and caring friends improves symptoms of depression	778 (48.8)
11	Mentors and support groups for the development of skills can have a positive influence on depressed persons	1392 (87.4)
12	Availability of opportunities for intellectual engagement within a person's community prevents depression	1231 (77.3)
13	Assurance of physical safety at one's place of residence reduces the incidence of depression	1086 (68.2)
14	Adequate physical exercise makes depressed persons feel all right	1016 (63.8)
15	Sufficient and regular sleeping pattern is not a necessary lifestyle for patients suffering from depression	642 (40.3)
16	Routine clinical examinations reduce the chances of suffering from teen depression	1025 (64.3)

Mean knowledge score = 62.1%

### Predictors of students' knowledge of risk factors for depression in young people

Table 4 revealed the result of the multivariate binary logistic regression to determine the demographic predictors of students' knowledge of risk factors linked with depression in young people. The result demonstrated that the course of study, year of study, contact with a depressed person, and personal experience of depression significantly predicted students' knowledge of the risk factors linked with depression in young people. Science-based students were about 1.67 times more likely to have a better knowledge compared to students in non-sciences (p < 0.001, AOR = 1.667, 95%CI = 1.336–2.073). First-year students were 64% less likely to have a good knowledge compared to the fifth-year students (p < 0.001, AOR = 0.360, 95%CI = 0.238–0.654). Students who have had contact with a depressed person have approximately 1.75 times higher odds of a better knowledge than those who have not (p < 0.001, AOR = 1.749, 95%CI = 1.365–2.242). Students with personal experience of depression had better knowledge compared to those who do not (p = 0.011, AOR = 1.355, 95%CI = 1.073–1.711).

**Table 4:** Multivariate logistic regression of students' demographic variables with knowledge of risk factors for depression in young people

Variable	AOR (95% CI), [Good = 1, Poor = 0]	P-value
**Course of study**		
Sciences	1.667 (1.336–2.073)	<0.001*
Non-sciences	Reference	
**Year of study**		
First-year	0.360 (0.238–0.654)	<0.001*
Second-year	0.410 (0.252–0.666)	<0.001*
Third-year	0.395 (0.223–0.581)	<0.001*
Fourth-year	0.492 (0.307–0.787)	0.003*
Fifth-year	Reference	
**Gender**		
Female	1.066 (0.861–1.320)	0.599
Male	Reference	
**Age (years)**		
16–20	0.402 (0.224–2.040)	0.336
21–25	0.645 (0.326–2.348)	0.415
26–30	0.711 (0.417–3.157)	0.557
≥ 31	Reference	
**Residence**		
Hostel	0.946 (0.765–1.169)	0.605
Off-campus	Reference	
**Contact with a depressed person**		
Yes	1.749 (1.365–2.242)	<0.001*
No	Reference	
**Ever visited a depressed person in** **psychiatric hospital**		
Yes	0.818 (0.581–1.151)	0.249
No	Reference	
**Knew a family member or friend** **who suffered from depression**		
Yes	0.683 (0.530–0.879)	0.056
No	Reference	
**Personal experience of depression**		
Yes	1.355 (1.073–1.711)	0.011*
No	Reference	

* Significant at p < 0.05; AOR = adjusted odd ratio; CI = confidence interval; Reference = 1

### Predictors of students' knowledge of protective factors against depression in young people

Table 5 showed the multivariate binary logistic regression of students' knowledge of protective factors against depression with their demographic characteristics. The significant predictors of students' knowledge were the course of study, year of study, and gender. Students in science-based courses had about 1.79 times better knowledge compared to non-science students (p < 0.001, AOR = 1.787, 95%CI = 1.433–2.230). Third-year students were 40% less likely to have a good knowledge than fifth-year students (p = 0.026, AOR = 0.606, 95%CI = 0.348–0.935). Female students had 1.38 times better knowledge compared to their male counterparts (p = 0.004, 95%CI = 1.112–1.714).

**Table 5:** Multivariate logistic regression of students' demographic variables with knowledge of protective factors against depression in young people

Variable	AOR (95% CI) [Good = 1, Poor = 0]	P-value
**Course of study**		
Sciences	1.787 (1.433–2.230)	<0.001*
Non-sciences	Reference	
**Year of study**		
First-year	0.571 (0.360–1.019)	0.059
Second-year	0.714 (0.432–1.182)	0.191
Third-year	0.606 (0.348–0.935)	0.026*
Fourth-year	0.786 (0.480–1.286)	0.337
Fifth-year	Reference	
**Gender**		
Female	1.380 (1.112–1.714)	0.004*
Male	Reference	
**Age (years)**		
16–20	0.303 (0.224–0.912)	0.336
21–25	0.411 (0.126–1.091)	0.415
26–30	0.851 (0.618–3.157)	0.557
≥ 31	Reference	
**Residence**		
Hostel	0.875 (0.707–1.084)	0.223
Off-campus	Reference	
**Contact with a depressed person**		
Yes	1.282 (0.997–1.649)	0.052
No	Reference	
**Ever visited a depressed person in** **psychiatric hospital**		
Yes	1.095 (0.773–1.551)	0.611
No	Reference	
**Knew a family member or friend who** **suffered from depression**		
Yes	0.761 (0.589–0.982)	0.136
No	Reference	
**Personal experience of depression**		
Yes	0.791 (0.854–1.374)	0.509
No	Reference	

* Significant at p < 0.05; AOR = adjusted odd ratio; CI = confidence interval; Reference = 1

## Discussion

The present study found that about forty-seven percent of the students had a good knowledge of the risk factors associated with depression in young people. Specifically, most of the students have correct knowledge that parental neglect, family conflict, low self-esteem, and stressful life events increase the likelihood of depression in young people. The knowledge of risks factors for depression would likely be beneficial in shaping students' lifestyle.^24^ However, a little more than a quarter of the students knew that female gender was at higher risk of suffering depression compared to the male folks. On the other hand, a higher proportion of students, about sixty percent had a good knowledge of the protective factors against depression.

The current study found that the course of study significantly predicted students' knowledge of risk and protective factors linked with depression in young people. Students in science-based courses had better knowledge compared to non-science students regarding the risk and protective factors linked with depression in young people. This finding was comparable to that reported in a study among university students in the United Kingdom.^25^ The study found a positive correlation between mental health literacy and previous exposure to lectures on mental disorders. In other words, students who were exposed to lectures on depression or related disorder had better knowledge than students who did not.^25^ Therefore, the curriculum content of the students in the current study could explain the difference in knowledge between science and non-science students. Generally, science-based students, for example, students in medical and health sciences appear more likely to undertake courses related to public and mental health. Also, a substantial proportion of science-based students in the present study were in disciplines such as medicine, health sciences, pharmacy, and other allied health courses.

The year of study was significantly associated with students' knowledge of risk and protective factors connected with depression. Higher year of study significantly predicted better knowledge of risk and protective factors related to depression in young people. It is expected that as students advance in their course of study, the higher the knowledge they might have acquired through formal lectures, experiences, and interactions with peers in the university community. Additionally, students in higher classes are perhaps older, more experienced, and have had more education than those at lower levels. Comparably, a previous related study on public knowledge of depression found that higher educational background was associated with better mental health literacy. 26 Also, another study among residents in Lagos, Nigeria reported that increasing level of education was associated with better knowledge of depression.^27^

Further, female students were found to have a better knowledge of protective factors against depression in young people. Higher female protective instinct could explain the difference in knowledge seen between male and female students. Another possible reason could be that female student are more likely to have experienced depression than their male counterparts.^28^ The later explanation was largely supported by the current study finding that students who have had personal experience of depression have a better knowledge of the risk factors related to depression. Similarly, students who have had previous contact with a depressed person had better knowledge of the risk factors associated with depression in young people. This finding was consistent with a previous study in Sweden, in which increased familiarity or contact with people with lived experience of depression was associated with a higher level of mental health literacy.^29^

The current study findings suggest the need for educational interventions aimed at addressing observed gaps in university students' knowledge of risk and protective factors linked with depression in young people. Students are faced with considerable challenges in the course of their university education, which could result in depression if not well handled. Health education on how to identify the potential risk of depression and how to avoid depression triggers would likely reduce the growing prevalence of depression and suicides among university students. In such training seminars or lectures, special considerations should be given to students in non-science disciplines and lower-level students. Furthermore, the management of universities should consider prioritizing the creation of a supportive learning environment by providing student mental health services to ease depression triggers among university students. A supportive learning environment would play a key role in improving students' mental wellbeing.

## Limitations

This study had a few limitations. First, this study was conducted in a single public university, hence the generalizability of its findings to other universities in Nigeria could not be ascertained. Secondly, the current study adopted a cross-sectional design, thus, could not determine a cause-effect relationship between students' demographic variables and their level of knowledge of risk and protective factors associated with depression in young people. Lastly, there was a possibility of social desirability and acquiescent response biases due to the use of self-reported instruments for data collection. However, to reduce or eliminate social desirability bias, we ensured the anonymity of respondents.

## Conclusion

Less than half of the students in this study had a good knowledge of the risk factors related to depression in young people. However, more than half of the students had a good knowledge of measures that protect young people from depression. Generally, students' level of knowledge was significantly predicted by the course of study, year of study, gender, previous contact with a depressed person, and personal experience of depression. Therefore, educational interventions in the university aimed at improving students' knowledge of depression, especially regarding factors that could trigger depression are strongly recommended. The management of universities should consider initiating student mental health services to mitigate depression tendencies among students.
